# Dichotomy between the humoral and cellular responses elicited by mRNA and adenoviral vector vaccines against SARS-CoV-2

**DOI:** 10.1186/s12916-022-02252-0

**Published:** 2022-01-25

**Authors:** Rahul Ukey, Natalie Bruiners, Hridesh Mishra, Pankaj K. Mishra, Deborah McCloskey, Alberta Onyuka, Fei Chen, Abraham Pinter, Daniela Weiskopf, Alessandro Sette, Jason Roy, Sunanda Gaur, Maria Laura Gennaro

**Affiliations:** 1 Public Health Research Institute, Rutgers New Jersey Medical School, ICPH Building, W250Q, 225 Warren Street, Newark, NJ 07103 USA; 2 Department of Medicine, Rutgers New Jersey Medical School, ICPH building, W250Q, 225 Warren Street, Newark, NJ 07103 USA; 3 Clinical Research Center, Rutgers Robert Wood Johnson Medical School, New Brunswick, NJ USA; 4 Global Tuberculosis Institute, Rutgers New Jersey Medical School, ICPH building, W250Q, 225 Warren Street, Newark, NJ 07103 USA; 5 Center for Infectious Disease and Vaccine Research, La Jolla Institute for Immunology, La Jolla, CA USA; 6 Department of Medicine, Division of Infectious Diseases and Global Public Health, University of California, San Diego, La Jolla, CA USA; 7 Department of Biostatistics and Epidemiology, School of Public Health, Rutgers University, Piscataway, NJ USA; 8 Department of Pediatrics, Rutgers Robert Wood Johnson Medical School, New Brunswick, NJ USA

**Keywords:** Ad26.COV2.S, BNT162b2, mRNA-1273, Antibody binding, Neutralizing antibodies, Antigen-specific B cells, Antigen-specific T cells

## Abstract

**Background:**

Protection from severe disease and hospitalization by SARS-CoV-2 vaccination has been amply demonstrated by real-world data. However, the rapidly evolving pandemic raises new concerns. One pertains efficacy of adenoviral vector-based vaccines, particularly the single-dose Ad26.COV2.S, relative to mRNA vaccines.

**Main body:**

We investigated the immunogenicity of Ad26.COV2.S and mRNA vaccines in 33 subjects vaccinated with either vaccine class 5 months earlier on average. After controlling for the time since vaccination, Spike-binding antibody and neutralizing antibody levels were higher in the mRNA-vaccinated subjects, while no significant differences in antigen-specific B cell and T cell responses were observed between the two groups.

**Conclusions:**

A dichotomy exists between the humoral and cellular responses elicited by the two vaccine classes. Testing only for humoral responses to compare the durability of SARS-CoV-2 vaccine-induced responses, as typically performed for public health and research purposes, is insufficient.

**Supplementary Information:**

The online version contains supplementary material available at 10.1186/s12916-022-02252-0.

With the COVID-19 pandemic still raging and new SARS-CoV-2 variants, such as Delta (B.1.617.2), exhibiting increased transmissibility [1], concerns have been raised about the efficacy of current vaccines in general as well as relative to each other. The SARS-CoV-2 vaccines that have received full approval or emergency use authorization by the US Food and Drug administration include the mRNA vaccines BNT162b2 (BioNTech-Pfizer) [2] and mRNA-1273 (Moderna) [3], which are administered in two doses, and the single-dose, adenoviral vector vaccine Ad26.COV2.S (Johnson and Johnson-Janssen) [4]. Comparisons of protective immune responses elicited by these vaccines have focused on neutralizing titers in the plasma [for example, [5, 6]]. Virus neutralization by plasma is critical to protect against viral infection, but understanding the efficacy and durability of vaccine-induced responses requires assessing both humoral and cellular adaptive immune responses elicited by vaccination.

Here, we used quantitative assays to compare antibody binding and neutralizing titers, antigen-specific B cell frequencies, and antigen-specific T cell responses in thirty-three participants with no history of SARS-CoV-2 infection, similarly divided between subjects fully vaccinated with mRNA vaccines (*n* = 16) or the adenoviral vector vaccine (*n* = 17). When we compared the two groups by age, gender, and co-morbidities, we found no difference in these variables except for the time elapsed since vaccination, which differed between the two groups (Table 1). Thus, the results of the immunological assays were adjusted by the time (in days) between full vaccine administration and blood collection for the study using linear regression.

**Table 1:** Demographics and clinical information of study participants, stratified by vaccine type

	Overall* (***n*** = 33)	J&J (***n*** = 17)	mRNA (***n*** = 16)	***p***
**Age (years)**	49.8 ± 15.6	52.3 ± 13.3	47.3 ± 17.7	0.066
**Gender**				
**Female**	17/33 (51%)	8/17 (47%)	9/16 (56%)	0.279
**Male**	16/33 (49%)	9/17 (53%)	7/16 (44%)	
**BMI (kg/m**^**2**^**)**	26.7 ± 5.3	27.4 ± 5.2	25.9 ± 5.4	0.413
**Race/ethnicity**				
**Asian**	10/33 (30%)	4/17 (24%)	6/16 (38%)	0.350
**Others**	1/33 (3%)	0/17 (0%)	1/16 (6%)	
**White**	22/33 (67%)	13/17 (76%)	9/16 (56%)	
**Hispanic or Latinx**	0/33 (0%)	0/17 (0%)	0/16 (0%)	–
**Comorbidities**				
**Yes**	10/33 (30%)	5**/17 (29%)	5**/16 (31%)	0.909
**No**	23/33 (70%)	12/17 (71%)	11/16 (69%)	
**Immunodeficiencies**				
**Yes**	3/33 (9%)	0/17 (0%)	3***/16 (19%)	0.061
**No**	30/33 (91%)	17/17 (100%)	13/16 (81%)	
**Days since vaccination**	168.2 ± 57.9	189.7 ± 62.8	145.2 ± 43.3	0.025

Data are presented as mean ± standard deviation or proportion (*n*/*N* (%))

*BMI* body mass index

*All study subjects were fully vaccinated [one-dose Johnson & Johnson (J&J) or two-dose mRNA vaccines]

**Hypertension (*n* = 6), obesity (*n* = 3), diabetes (*n* = 2), asthma (*n* = 2), and coronary artery disease (*n* = 1) (some conditions were concurrent)

***Neutropenia (*n* = 1), rheumatoid arthritis (*n* = 1), and use of corticosteroids (*n* = 1)

## Materials and methods

### Ethics, consent, and permission

Thirty-three subjects who received either mRNA vaccines (*n* = 16) or the adenoviral vector vaccine Ad26.COV2.S (*n* = 17) were enrolled on August 9–10, 2021, at the Rutgers Robert Wood Johnson Medical School, New Brunswick, NJ, USA. All participants self-reported no history of SARS-CoV-2 infection and date of vaccination and consented to blood draws as well as collection of demographic data. All study activities were approved by the Rutgers Institutional Review Board (Pro2020000655).

### Biosafety protocols

All work involving blood products from SARS-CoV-2-infected subjects was performed in a biosafety level 2+ (BSL-2+) laboratory-utilizing protocols approved by the Rutgers Institutional Biosafety Committee. All plasma samples were heat-inactivated at 56 °C for 60 min before testing. Work involving live SARS-CoV-2 was performed in a biosafety level 3 (BSL-3) laboratory-utilizing protocols approved by the Rutgers Institutional Biosafety Committee.

### Antibody binding by enzyme-linked immunosorbent assay (ELISA)

Antibody binding was performed by ELISA platform utilizing SARS-CoV-2 receptor-binding domain (RBD) of the Spike protein as solid-phase antigen and standard operating procedures, as described [7]. Each sample was tested in duplicate. End-point titers were calculated using an established cutoff [7] and background-subtracted data.

### Cell lines

Vero E6 were obtained from the American Type Culture Collection (ATCC), Manassas, USA; HeLa cells stably expressing ACE2 (HeLa-ACE2) were obtained from Dennis Burton at the Scripps Research Institute [8]. All cell lines were maintained in high-glucose Dulbecco’s modified Eagle’s medium (DMEM; Corning, Manassas, USA) supplemented with 10% fetal bovine serum (FBS; Seradigm, Radnor, USA), 2 mM l-glutamine, and 1% penicillin/streptomycin (Corning, Manassas, USA) and incubated in humidified atmospheric air containing 5% CO_2_ at 37 °C.

### SARS-CoV-2 virus

The virus stock of mNeonGreen (mNG) SARS-CoV-2 was obtained from Pei-Yong Shi at the University of Texas Medical Branch at Galveston. The virus stock was produced using the virus isolate of the first patient diagnosed in the USA, in which the ORF7 of the viral genome was replaced with the reported mNG gene [9]. Propagation of viral stocks was performed with Vero E6 cells using 2% FBS. The virus titers were determined by standard plaque assay utilizing Vero E6 cells [10] and recorded as plaque-forming units per milliliter (PFU/mL).

### SARS-CoV-2 neutralization assay

HeLa-Ace2 cells were seeded in 96-well black optical-bottom plates at a density of 1 × 10^4^ cells/well in FluoroBrite DMEM (Thermo Fisher Scientific, Waltham, USA) containing 4% FBS (Seradigm), 2 mM l-glutamine, and 1% penicillin/streptomycin (Corning, Manassas, USA), and incubated overnight at 37 °C with 5% CO_2_. On the following day, each sample was subjected to two-fold serial dilution in DMEM without FBS and incubated with mNG SARS-CoV-2 at 37 °C for 1.5 h. The virus-plasma mixture was transferred to 96-well plates containing Hela-Ace2 cells at a final multiplicity of infection (MOI) of 0.25 (viral PFU:cell). For each sample, the starting dilution was 1:20 and the final dilution of 1:10,240. After incubating infected cells at 37 °C for 20 h, mNG SARS-CoV-2 fluorescence was measured using a Cytation™ 5 reader (BioTek, Winooski, USA). Each sample was tested in duplicate. Relative fluorescent units were converted to percent neutralization by normalizing the sample treatment to non-sample treatment controls and plotting the data with a nonlinear regression curve fit to determine the titer neutralizing 50% of SARS-CoV-2 fluorescence (NT_50_).

### PBMC isolation and storage

Peripheral blood mononuclear cells (PBMC) were isolated by Ficoll density gradient centrifugation (Ficoll-Paque, GE Healthcare, Uppsala, Sweden), as described [11], cryopreserved in liquid nitrogen in FBS containing 10% dimethyl sulfoxide (DMSO; Thermo Fischer Scientific, Waltham, MA, USA), and stored until use.

### RBD-specific B cell immunostaining

To form RBD tetramers, biotinylated RBD (BioLegend, San Diego, CA, USA) was mixed in separate tubes with streptavidin-conjugated with Alexa Fluor 647 or BV421 (BD Biosciences, San Jose, CA, USA) at a 4:1 molar ratio for 1 h at 4 °C. PBMCs were stained with fixable viability stain 780 (BD Biosciences, Franklin Lakes, USA), incubated for 10 min with human Fc receptor blocking reagent (BD Biosciences, Franklin Lakes, NJ, USA), and then stained with the two fluorescent RBD tetramers, antibodies to CD19-BV700 (HIB19, Bio Legend, San Diego, CA, USA) and CD20-PE-CF594 (2H7, BD Biosciences, San Jose, CA, USA) to detect RBD-specific B cells, and APC-Cy7-labeled antibodies against CD3 (UCHT1), CD4 (OKT4), CD14 (C1D3), and CD16 (CD16) (all from Thermo Fisher Scientific, Waltham, MA, USA) to eliminate non-B cells. Samples were incubated for 30 min on ice in the dark to allow for antibody binding, washed twice with FACS buffer (2% FBS in PBS), fixed for 20 min with 4 percent paraformaldehyde (PFA; Thermo Fisher Scientific, Waltham, MA, USA), and stored at 4 °C overnight. At least 500,000 events were collected per sample utilizing a Fortessa X-20 flow cytometer (BD Biosciences, San Jose, CA, USA). After removing dead and non-B cells, B cells were separated as CD19^+^CD20^+^, and the frequency of B cells positive for both RBD tetramers was determined by using the Flow Jo software (Flow Jo LLC, USA).

### SARS-CoV-2-derived peptides

A megapool (MP_S) of 253 15-mer synthetic peptides overlapping by 10-residues that cover the entire spike (S) antigen was generated based on predicted SARS-CoV-2 CD4 T cell epitopes, as previously reported [12, 13].

### IFNγ release assay

PBMC were washed in pre-warmed RPMI 1640 supplemented with 2 mM l-glutamine, 10% FBS, 100 U/ml penicillin, and 100 μg/ml streptomycin (complete RPMI) (all from Corning cellgro, Manassas, VA, USA), seeded in a 48-well cell culture plate at a density of 1 × 10^4^ cells/well in complete RPMI, and stimulated with 1μg/ml of the SARS-CoV-2 MP_S peptide pool or 0.1% DMSO (vehicle control). Cell culture plates were incubated for 24 h at 37 °C in a 5% CO_2_-humidified atmosphere. Each sample was tested in duplicate. As a positive control, two wells per sample were treated with a mixture of 25 ng/ml phorbol 12-myristate 13-acetate (PMA) (Sigma-Aldrich, St. Louis, MO, USA) and 0.5 μM ionomycin calcium salt (Enzo Life Sciences, Farmingdale, CT, USA) for 2 h. Supernatants were collected, and levels of IFNγ in supernatants were assayed using a commercial Human IFNγ ELISA kit (BD Biosciences, San Jose, CA), according to the manufacturer’s instructions.

### Statistical analysis

Baseline demographic and other variables were tested using a two-sample proportion test and Student’s *t*-test. All flow cytometry data were analyzed with the FlowJo v12 software (FlowJo LLC, Ashland, OR, USA). Measurements from all immunological assays were compared between the two study groups using the Mann-Whitney *U* test. Linear regression was performed to assess the dependency of immunological measurements on the type of vaccination while adjusting for time (days) elapsed since vaccination and adjusting for age. The correlation was analyzed using Spearman’s rank correlation. For all tests, *p* < 0.05 was considered significant. Statistical analyses were performed with Stata (version 17, StataCorp LLC, College Station, USA) and GraphPad Prism version 8.4 (Graph Pad Software Inc., La Jolla, USA).

#### Antibody binding and neutralization

**Fig. 1:**
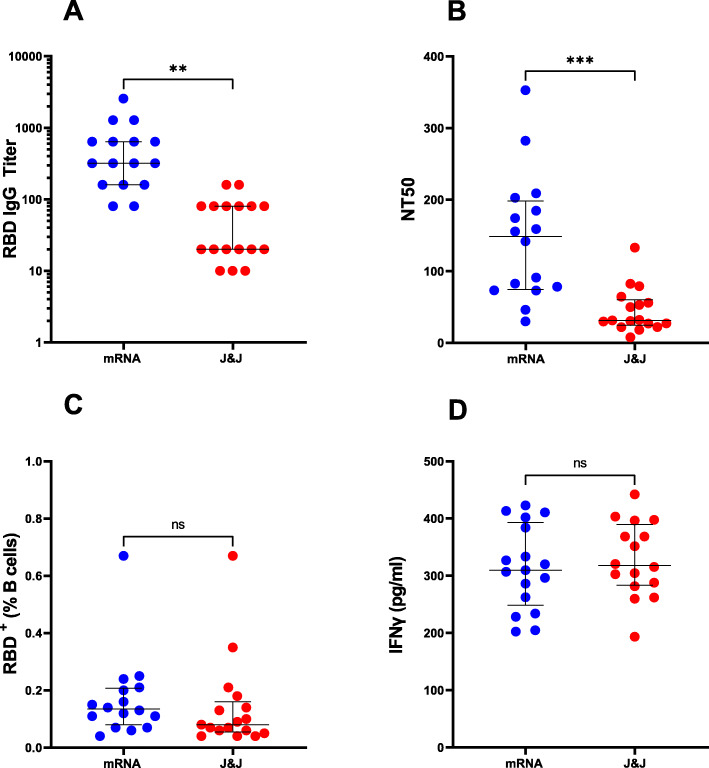
Humoral and cellular responses elicited by mRNA and adenoviral vector-based COVID-19 vaccines. Each circle indicates one subject. Blue circles represent subjects who received two doses of mRNA vaccine (*n* = 16), and red circles represent subjects who received the adenoviral vector-based (J&J) vaccine (*n* = 17). The dot plots show **A** anti-RBD IgG antibody levels, **B** neutralizing titers expressed as NT50 (reciprocal dilution of plasma yielding 50% neutralization of live SARS-CoV-2 virus), and **C** frequency (%) of RBD-specific B cells. B cells (CD19^+^CD20^+^) were analyzed for RBD specificity utilizing dual fluorescent labeling of RBD tetramers. **D** IFNγ (pg/ml) production by antigen-specific T cells. PBMCs from each subject were stimulated with a megapool of overlapping synthetic peptides (15-mers) covering the entire Spike (S) antigen. Supernatants were collected after 24 h of stimulation. In all panels, the solid black lines represent the median and interquartile range. Statistical analyses were conducted by the Mann-Whitney *U* test for unpaired samples (**p ≤ 0.01; ***p ≤ 0.001; ns, non-significant, *p* > 0.05)

All vaccines express the full-length SARS-CoV-2 Spike protein [2–4]. We analyzed the plasma of all subjects for IgG antibody binding the receptor-binding domain (RBD) of the S1 subunit of the SARS-CoV-2 Spike protein and for neutralizing antibodies. We chose RBD as the target antigen of the antibody response, because the neutralizing activity of plasma is largely directed against RBD, as shown by us and others [14–16]. The virus neutralization activity of plasma was measured with an assay utilizing replication-competent SARS-CoV-2 virus. We found that both Ab binding and neutralizing titers were higher in the mRNA-vaccinated group relative to adenoviral vector vaccinees (Fig. 1AB). The differences between the groups were statistically significant after adjusting for days since vaccination (Table 2).

**Table 2:** Estimates of the mean difference in each measurement between mRNA and J&J (reference) vaccinees from a linear regression, adjusted for time since vaccination

Variable	Estimate (95% CI)	Standard error	***p***
**Anti-RBD IgG titers**	519.9 (169.1, 870.8)	171.8	0.0051
**NT50**	99.1 (47.9, 150.3)	25.1	0.0004
**RBD**^**+**^ **(%B cells)**	0.036 (− 0.084, 0.156)	0.059	0.55
**IFNγ (pg/ml)**	35.7 (− 15.0, 86.4)	24.8	0.16

*CI* confidence interval, *RBD* receptor-binding domain, *IgG* immunoglobulin G, *NT50* neutralization titer at 50% inhibition

#### RBD+ B cell frequencies

The levels of specific antibodies in the circulation physiologically decrease with time elapsed since exposure to antigen [14, 17, 18]. Thus, assessing the durability of vaccine-induced responses and protection from occurrence and clinical severity of breakthrough infection requires the evaluation of antigen-specific B cells and T cells. To analyze the memory B cell response elicited by the vaccine, we enumerated RBD-specific B cells (RBD-tetramer-positive CD19^+^CD20^+^) utilizing dual RBD tetramer staining for high specificity and multi-color flow cytometry (the gating strategy is shown in Additional file 1: Fig. S1). We found that the differences in RBD-specific B cell frequencies between the mRNA- and adenoviral vector-vaccinated subjects were not statistically significant (Fig. 1C and Table 2).

#### Interferon gamma (IFNγ) release assay

A straightforward method to assess antigen-specific T cells is measuring the production of T cell cytokines, such as IFNγ, by peripheral blood mononuclear cells (PBMC) stimulated ex vivo with peptides representing T cell epitopes, as performed for many infectious and non-infectious conditions (for example, [19]). We used a previously described peptide pool containing hundreds of overlapping 15-meric peptides spanning the Spike protein (MP_S) [12] for PBMC stimulation and detection of IFNγ release by ELISA. This assay showed no significant differences between the two groups of vaccinees (Fig. 1D and Table 2).

#### Correlation between humoral and cellular responses

To assess the complementarity between humoral and cellular responses, we calculated the correlations among the results of each immunological assay in a pairwise manner. As expected, anti-RBD antibody-binding and plasma-neutralizing activity highly correlated with each other (corr = 0.87—where 1 is the maximum possible value), since the neutralizing activity of the plasma mostly resides in the RBD-specific antibodies, as noted above. In contrast, the correlation between cellular and humoral assay and between the two cellular assays with each other was very low (corr < 0.15), indicating that these immunological readouts are independent of each other.

#### Effect of age on the vaccine response

Since the two vaccine groups showed a non-significant trend for age mean (*p* = 0.06) (Table 1) and age may affect the response to SARS-CoV-2 vaccination [20], we calculated the correlations between age and either antibody responses or cellular responses. We found a weak-moderate correlation between the age and antibody response [Age and IgG (corr = − 0.25, *p* = 0.16); age and neutralizing antibodies (corr = − 0.23, *p* = 0.20)] and a very weak correlation between age and cellular response [age and B cells (corr = 0.002, *p* = 0.99); age and T cells (corr = − 0.09, *p* = 0.60)]. To assess whether age is an explanation for the differences in antibody response between the vaccine groups, we adjusted for age in a linear regression model. The resulting adjusted difference in means was − 477.7 (*p* = 0.01) for anti-RBD IgG and − 94.2 (*p* < 0.001) for neutralizing antibodies. Thus, our conclusions did not change when we controlled for age.

In conclusion, mRNA vaccination results in higher levels of circulating binding and neutralizing antibodies than the adenoviral vector counterpart (at least in the timeframe of our study, i.e., 5 months after vaccination on average), while the antigen-specific cellular responses to the two vaccine classes show no significant differences. It is noted that a limitation of our study is the small sample size (*n* = 33 in the two groups). The different antibody levels are likely due to the single-dose administration of the adenoviral vector-based vaccine vs the two-dose protocols applied with the mRNA vaccines. Indeed, antibody titers measured after the second dose of mRNA vaccines are higher than after the first dose [5, 21], and the antibody responses to a booster dose are more vigorous than those elicited by primary vaccination protocols [22, 23]. Thus, circulating antibody levels increase with the number of doses of our current SARS-CoV-2 vaccines. Since vaccine-induced neutralizing antibodies are highly correlated with immune protection from symptomatic infection [24, 25]—a correlation supported by murine studies [26], our data imply that a booster dose of the Ad26.COV2.S is particularly advisable, especially in the face of the global rise of COVID-19 morbidity due to the highly infectious SARS-CoV-2 Delta variant [1]. Indeed, the administration of a second vaccine dose of Ad26.COV2.S predictably induces a stronger antibody response than the primary vaccination, as per interim data by the manufacturer [27].

Our results may indirectly help explain why protection from symptomatic infection afforded by SARS-CoV-2 vaccination tends to wane while vaccine efficacy in preventing hospitalization and death persists [28, 29]. The observed dichotomy between humoral and cellular immune responses elicited by the two SARS-CoV-2 vaccine classes is consistent with different trajectories of decay of the humoral and cellular responses, with circulating antibody levels decreasing more rapidly than antigen-specific memory responses, which tend to last longer. One may postulate that humoral immunity provides a “ready-to-go” response to reinfection that limits viral replication and the consequent development of symptoms. In contrast, memory immune responses, which require longer to express protective functions even in vaccinated individuals, may defend against severe disease and hospitalization. If so, rapidly decaying humoral responses vis-à-vis persisting cellular responses to vaccination might underlie the loss of protection from symptomatic infection while defense from severe consequences of infection, such as hospitalization and death, is still afforded. Future studies on the durability of vaccine-induced protection, the protective effects of booster doses, and the underlying immunological mechanisms will test our proposition.

## Supplementary Information

**Additional file 1: Figure S1.** Gating strategy for SARS-CoV-2 S1 RBD-specific memory B cells. (A) Physical parameters; (B) Exclusion of dead cells and non-B cells (CD14^+^, CD3^+^, CD4^+^, CD16^+^); (**C**) CD19^+^CD20^+^ B cells were further gated to distinguish (**D**) RBD-specific B cells based on dual labeling in the same staining tube with two fluorescent RBD tetramers separately conjugated with Alexa Fluor 647 and BV421.

## Data Availability

Data are available on reasonable request to the corresponding author due to privacy/ethical restrictions.

## Abbreviations

ACE2: Angiotensin-converting enzyme-2
Ad26.COV2.S: Johnson and Johnson-Janssen adenoviral vector
ATCC: American Type Culture Collection Center
BNT162b2: BioNTech-Pfizer mRNA vaccine
BSL-2+: Biosafety level 2+
BSL-3: Biosafety level 3
DMEM: High-glucose Dulbecco’s modified Eagle’s medium
DMSO: Dimethyl sulfoxide
ELISA: Enzyme-linked immunosorbent assay
FBS: Fetal bovine serum
HeLa-ACE2: HeLa cells stably expressing ACE2
IFNγ: Interferon gamma
mNG: mNeonGreen
MOI: Multiplicity of infection
MP_S: Overlapping 15-meric peptides spanning the spike protein
mRNA-1273: Moderna mRNA vaccine
NT50: Titer neutralizing 50% of SARS-CoV-2 fluorescence
PBMC: Peripheral blood mononuclear cells
PFA: Paraformaldehyde
PFU/ml: Plaque forming units per milliliter
PMA: Phorbol 12-myristate 13-acetate
RBD: Receptor-binding domain
RBD-specific B cells: RBD-tetramer-positive CD19^+^CD20^+^
S: Spike protein
SARS-CoV-2: Severe acute respiratory syndrome coronavirus-2
