# Supplementary figures and images for: Dichotomy between the humoral and cellular responses elicited by mRNA and adenoviral vector vaccines against SARS-CoV-2
Source: BMC Med. 2022 Jan 25;20:32. doi: 10.1186/s12916-022-02252-0 (PMC8786593; doi:10.1186/s12916-022-02252-0)

**Figure S1**

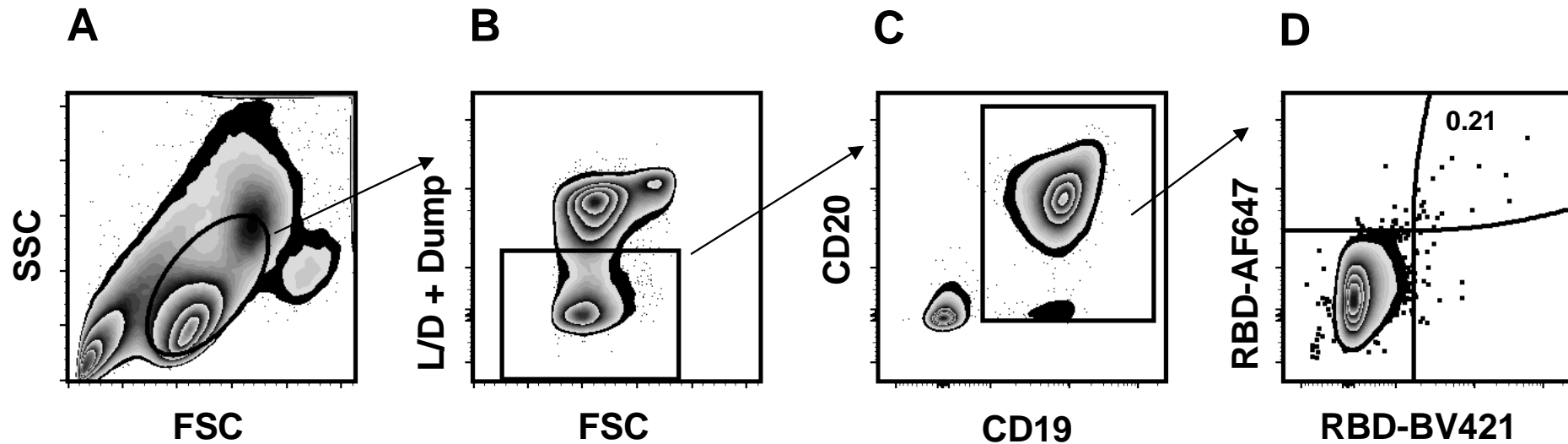

Supplement: Supplementary file 1 — Additional file 1: Figure S1. Gating strategy for SARS-CoV-2 S1 RBD-specific memory B cells. (A) Physical parameters; (B) Exclusion of dead cells and non-B cells (CD14+, CD3+, CD4+, CD16+); (C) CD19+CD20+ B cells were further gated to distinguish (D) RBD-specific B cells based on dual labeling in the same staining tube with two fluorescent RBD tetramers separately conjugated with Alexa Fluor 647 and BV421. [file 12916_2022_2252_MOESM1_ESM.pdf]
